# Correlation between tangential distortion of the outer retinal layer and metamorphopsia in patients with epiretinal membrane

**DOI:** 10.1007/s00417-021-05077-4

**Published:** 2021-01-16

**Authors:** Daiki Sakai, Seiji Takagi, Yasuhiko Hirami, Makoto Nakamura, Yasuo Kurimoto

**Affiliations:** 1 Department of Ophthalmology, Kobe City Eye Hospital, 2-1-8 Minatojima Minamimachi, Chuo-ku, Kobe-shi, Hyogo 650-0047 Japan; 2 Department of Ophthalmology, Kobe City Medical Center General Hospital, Kobe, Japan; 3 Department of Surgery, Division of Ophthalmology, Kobe University Graduate School of Medicine, Kobe, Japan; 4 Department of Ophthalmology, Toho University Graduate School of Medicine, Tokyo, Japan

**Keywords:** Epiretinal membrane, Metamorphopsia, Optical coherence tomography, Outer nuclear layer, Outer retinal layer

## Abstract

**Purpose:**

To evaluate tangential morphological changes in the outer retina and assess their correlation with the degree of metamorphopsia in patients with idiopathic epiretinal membrane (ERM).

**Methods:**

This retrospective study included patients with idiopathic ERM who underwent vitrectomy between January 2018 and December 2019. We evaluated the preoperative examination results. Using cross-sectional spectral-domain optical coherence tomography (OCT) images along the horizontal/vertical meridian through the fovea, we defined a new parameter, tangential displacement (TD), as the tangential component of the position vector of the distorted outer nuclear layer caused by ERM. Visual function measurements included M-CHARTS results (vertical/horizontal metamorphopsia score [MV/MH]) and best-corrected visual acuity (BCVA). The correlations among the OCT parameters including TD and central foveal thickness (CFT) with visual function measurements were determined.

**Results:**

Overall, 78 eyes of 76 patients (49 females; mean age, 67.9 [± standard deviation, 7.5 years]) were included. The mean horizontal TD was 24.0 ± 73.9 μm, which was significantly different from 0 (*p* = 0.005). The mean vertical TD was 6.0 ± 76.2 μm, which was not significantly different from 0. The absolute value of horizontal TD was significantly correlated with MV (*r* = 0.513, *p* < 0.01) and MH (*r* = 0.423, *p* < 0.01). The absolute value of vertical TD was also significantly correlated with MV (*r* = 0.274, *p* = 0.02) and MH (*r* = 0.413, *p* < 0.01). However, neither value was significantly correlated with BCVA. Multiple regression analysis showed that the horizontal absolute TD was an independent factor associated with both MV (β = 0.635, *p* < 0.001) and MH (β = 0.259, *p* = 0.048).

**Conclusion:**

We found that ERM tended to distort the outer retinal layer toward the temporal side of the fovea. The tangential distortion of this layer was associated with the degree of metamorphopsia, suggesting that misalignment of parafoveal photoreceptors causes metamorphopsia in patients with ERM.

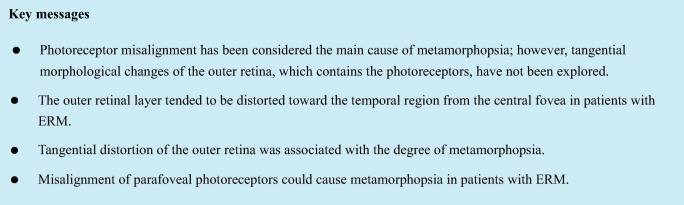



## Introduction

Idiopathic epiretinal membrane (ERM) is a relatively common retinal condition characterized by the formation of translucent membrane tissue on the surface of the neurosensory retina. Membrane contraction leads to macular distortion, resulting in metamorphopsia and reduced visual acuity (VA) [1]. Metamorphopsia is an important symptom experienced by patients with ERM, and it has been reported that metamorphopsia, rather than VA, strongly influences the vision-related quality of life in these patients [2].

The development of spectral-domain optical coherence tomography (SD-OCT) has enabled a more detailed evaluation of the retinal microstructure layer by layer, because of its high resolution and high scanning speed. Currently, SD-OCT is the most powerful tool for evaluating ERM.

Several studies have been performed to assess the correlation between changes in retinal microstructure and metamorphopsia using SD-OCT. Previous investigations have focused on measuring the thickness of individual retinal layers. The thickness of the inner nuclear layer (INL) [3–7], inner retinal layer (vitreous surface to the outer border of the INL) [8], outer nuclear layer (ONL) plus outer plexiform layer (OPL) [6], and central foveal thickness (CFT) [4, 6] have been reported to be associated with metamorphopsia. The INL thickness seems to be the most convincing factor indicating the severity of metamorphopsia. Ichikawa et al. reported that ERM contraction leads to displacement of Müller cells, which are found in the INL, from their original locations. The authors suggested that irregular photoreceptor stimulation through distorted Müller cells may cause metamorphopsia [7]. Since the tangential traction force of ERM primarily affects the inner retina, it is reasonable that the inner retina is more vulnerable than the outer retina in patients with ERM. However, photoreceptor misalignment itself has been considered the main cause of metamorphopsia [9].

Measurements of retinal layer thickness naturally evaluate the effects of centripetal force transmitted from the tangential traction force of the ERM; however, these measurements seem insufficient to evaluate the alignment of retinal cells. Therefore, to investigate the indicator of metamorphopsia, we considered that the tangential morphological change of the outer retina which contains the photoreceptors may be the preferable object.

The purpose of this study was to evaluate anatomical changes in the outer retinal layer, especially in the tangential direction, and to assess their correlation with the degree of metamorphopsia in patients with idiopathic ERM. The primary objective was to quantify tangential morphological changes in the outer retina using SD-OCT. The secondary objective was to evaluate the relationship between visual function and the degree of tangential morphological changes in the outer retina.

## Methods

### Study design

This retrospective study was performed according to the Declaration of Helsinki and was approved by the medical ethics committee of the Kobe City Medical Center General Hospital (Kobe, Japan). The committee waived the requirement for informed consent for this observational study involving the use of medical records because the confidentiality of patient data was maintained.

### Patients

Medical chart review was performed for the 124 eyes of 121 patients with idiopathic ERM who underwent pars plana vitrectomy from January 2018 to December 2019 at the Kobe City Eye Hospital. ERM was defined as a translucent membrane tissue on the surface of the internal limiting membrane with macular thickening detected on biomicroscopy and spectral domain OCT images. The eyes with other retinal confounding diseases including glaucoma (*n* = 14) or age-related macular degeneration (*n* = 2) and a long axial length more than 27 mm (*n* = 8) were excluded. We also excluded eyes with pseudohole-type (fovea-sparing) ERM [10] (*n* = 22) because we focused on foveal microstructure changes caused by ERM in this study.

### Measurements

**Fig. 1:**
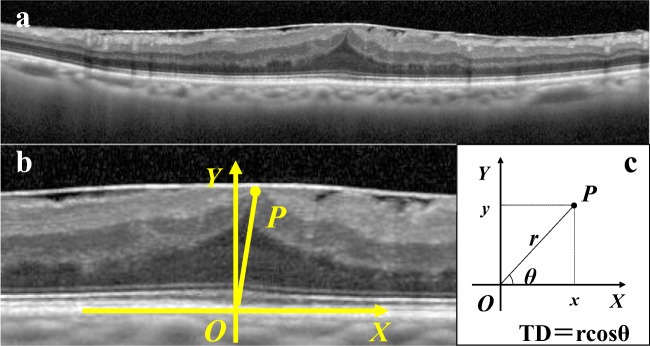
Measurement of tangential displacement (TD) on a spectral-domain optical coherence tomography (SD-OCT) image. (**a**) Cross-sectional SD-OCT image along the horizontal meridian through the fovea in the left eye. (**b**) Magnified image of **(a)** at the level of the central fovea. The point on the retinal pigment epithelium at the central fovea was identified as the coordinate origin (O) by referring to the foveal bulge. The apex of the outer nuclear layer was identified as the point P. (**c**) Graphing in the polar coordinate system. The value “r” was measured as the length from point P to the origin, and the value “θ” was measured as the angle between the positive x-axis of coordinate and a ray going through point P. The TD value was calculated by using the following equation: TD = rcosθ

Preoperative VA, metamorphopsia, and OCT images were evaluated. Best-corrected VA (BCVA) was obtained using Landolt C charts and converted to the logarithm of the minimum angle of resolution (logMAR) equivalent for statistical comparisons. The degree of metamorphopsia was quantified using M-CHARTS (Inami Co., Tokyo, Japan), which is a simple method of measuring a patient’s subjective metamorphopsia score horizontally and vertically [11]. M-CHARTS consists of one straight line and 19 dotted lines with dot intervals changing from 0.2 to 2.0° of the visual angle. In short, the patients obtained the minimum visual angle to recognize the dot shift at distance of 30 cm vertically (vertical metamorphopsia score [MV]) and horizontally (horizontal metamorphopsia score [MH]). SD-OCT images were obtained using Spectralis (Heidelberg Engineering, Heidelberg, Germany). Based on SD-OCT image measurements, we defined a new parameter of anatomical changes in the outer retinal layer that provides an indication of tangential photoreceptor displacement. This new parameter, called tangential displacement (TD), was used to describe the tangential component of the position vector of the distorted ONL apex caused by ERM. Preoperative cross-sectional SD-OCT images along the horizontal or vertical meridian through the fovea were evaluated. Each image was regarded as a coordinate system. The point on the retinal pigment epithelium (RPE) at the central fovea was identified as the coordinate origin by referring to the foveal bulge. If the foveal bulge was absent, the origin was identified by comprehensively considering the fixation point or the point of maximum ONL thickness and minimum inner retinal layer thickness. Then, the apex of the distorted ONL was identified as the innermost border of the ONL, at the thickest point of the ONL. Given that point P was the apex of the distorted ONL, the value “r” was measured as the length from point P to the origin, and the value “θ” was measured as the angle between the positive *x*-axis of coordinate (tangent to the RPE surface at origin) and a ray going through point P. The *x*-coordinate of point P was defined as the TD value using the following equation: TD = rcosθ (Fig. 1). The *x*-axis was always set such that the positive direction was consistent with the direction toward the temporal side from the fovea in the OCT image along the horizontal meridian (horizontal OCT image), i.e., the horizontal line to the right of the origin was the positive *x*-axis in the horizontal OCT image of the left eye, and the horizontal line to the left of the origin was the positive *x*-axis in the horizontal OCT image of the right eye. Because the horizontal OCT images of the left and right eyes have opposite orientations, this irregular method was adopted for the analysis of TD distribution. A positive horizontal TD value indicated that ONL was distorted in the temporal direction, whereas a negative horizontal TD value indicated distortion in the nasal direction. Similarly, a positive vertical TD value indicated that ONL was distorted in the superior direction, and a negative vertical TD value indicated distortion in the inferior direction. The absolute value of TD (absolute TD) was used to assess the relationship between the degree of TD and visual function. Additionally, we measured central foveal thickness (CFT) to describe the centripetal changes of retina. The CFT was defined as the length from the vitreoretinal interface and to the inner border of the RPE. The average of CFT values obtained from horizontal and vertical OCT images was used to assess the relationship with visual function. The length was measured using the “caliper” function of the Heidelberg instrument. The angle was measured using Image J software (National Institute of Health, Bethesda, MD, USA). Both the length and angle measurements were performed twice by one observer (D.S.), and the average of each measurement was used to calculate the TD and CFT value. The observer was masked to the visual function of patients at the time of SD-OCT image measurements. Images were adjusted to an aspect ratio of 1:1 mm in all cases.

### Statistical analysis

All statistical analyses were conducted using the SPSS statistical software package (version 25; SPSS Inc., Chicago, IL, USA). The one-sample *t* test was used to determine whether the horizontal or vertical TD followed a normal distribution with a mean equal to zero. The absolute TD value was used to assess the relationship between the degree of TD and visual function. Spearman’s rank correlation coefficient was used to determine correlations among the OCT parameters (horizontal or vertical absolute TD and CFT) with M-CHARTS scores and BCVA as univariate analyses. Then, multiple regression analysis was performed to determine correlations among the OCT parameters with M-CHARTS scores. Continuous variables were compared using the Mann–Whitney *U* test. *p* ≤ 0.05 was considered statistically significant.

## Results

A total of 78 eyes of 75 patients (49 female) with idiopathic fovea-attached ERM were included. The patients’ clinical data are summarized in Table 1. The mean (± standard deviation) age was 67.9 years (± 7.5 years). Seventy-four eyes were phakic preoperatively. Both nine eyes of foveal bulge were absent in horizontal and vertical OCT images, respectively. Visual function measurement results at preoperative examination were as follows. The mean logMAR BCVA was 0.16 (± 0.19) with a range of − 0.18 to 0.70, the mean MV was 0.79 (± 0.62) with a range of 0 to 2.0, and the mean MH was 0.82 (± 0.60) with a range of 0 to 2.0. Twelve patients had the MV value of 0 and 10 patients had the MH value of 0. Among them, 6 patients did not have metamorphopsia (both MV and MH were 0) preoperatively, and their indications for surgery were visual acuity impairment.

**Table 1:** Demographic data of the 78 eyes of 76 patients

	76 patients
Age (years), mean ± SD	67.9 ± 7.5
Female, *n* (%)	49 (64.5)
	78 eyes
Lens status	
Phakia, *n* (%)	74 (94.9)
Pseudophakia, *n* (%)	4 (5.1)
Visual function measurements	
LogMAR BCVA, mean ± SD	0.16 ± 0.19
Range	− 0.18 to 0.70
MV, mean ± SD	0.79 ± 0.62
Range	0 to 2.0
MH, mean ± SD	0.82 ± 0.60
Range	0 to 2.0
OCT measurements	
Horizontal TD (μm), mean ± SD	24.0 ± 73.9
Range	− 109.8 to 226.6
Vertical TD (μm), mean ± SD	6.0 ± 76.2
Range	− 170.5 to 184.1
Absolute value of horizontal TD (μm), mean ± SD	60.5 ± 48.4
Absolute value of vertical TD (μm), mean ± SD	61.3 ± 45.1
CFT (μm), mean ± SD	458.0 ± 85.1
Range	287.3 to 682

*SD* standard deviation, *BCVA* best-corrected visual acuity, *MV* vertical metamorphopsia score, *MH* horizontal metamorphopsia score, *OCT* optical coherence tomography, *TD* tangential displacement, *CFT* central foveal thickness

**Fig. 2:**
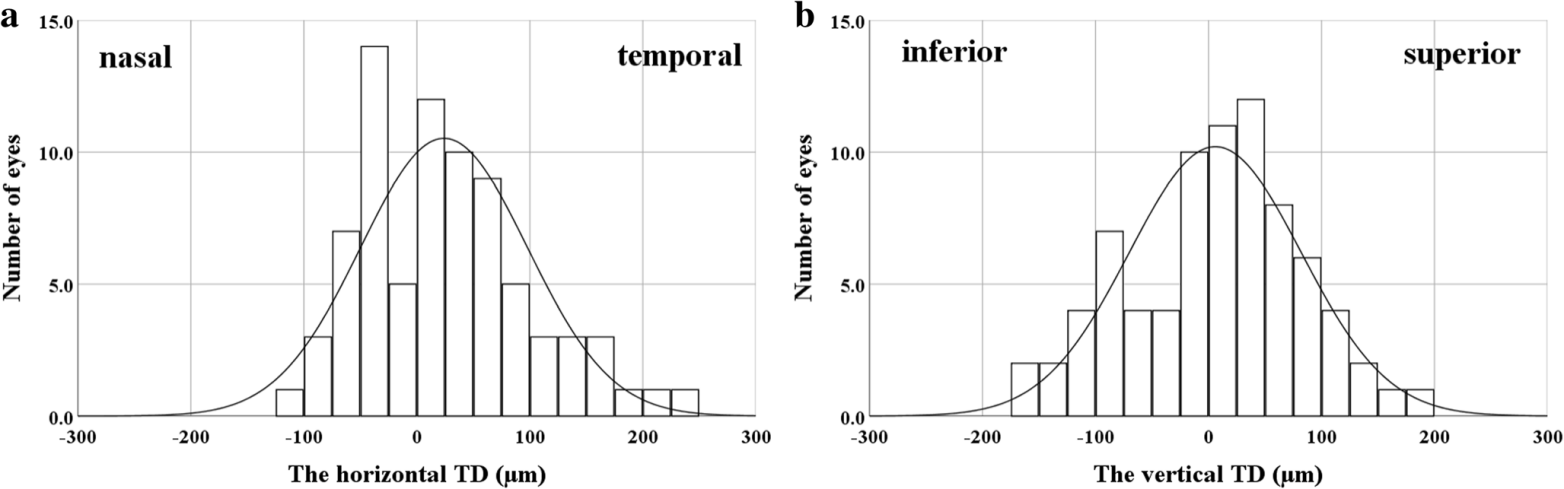
Histograms showing the distribution of the horizontal (**a**) or vertical (**b**) “tangential displacement (TD)” at preoperative examination

Figure 2 shows the distribution of the horizontal or vertical TD at preoperative examination. The mean horizontal TD was 24.0 μm (± 73.9 μm), which was significantly different from 0 (one-sample *t* test, *p* = 0.005, 95% confidence interval [CI]: 7.3–40.7). The mean vertical TD was 6.0 μm (± 76.2 μm), which was not significantly different from 0 (one-sample *t* test, *p* = 0.49, 95% CI: − 11.2 to 23.1). The mean horizontal absolute TD was 60.5 μm (± 48.4 μm), and the mean vertical absolute TD was 61.3 μm (± 45.1 μm). There was no significant difference between the horizontal and vertical absolute TD (Mann-Whitney *U* test, *p* = 0.76). The mean CFT was 458.0 μm (± 85.1 μm).

**Fig. 3:**
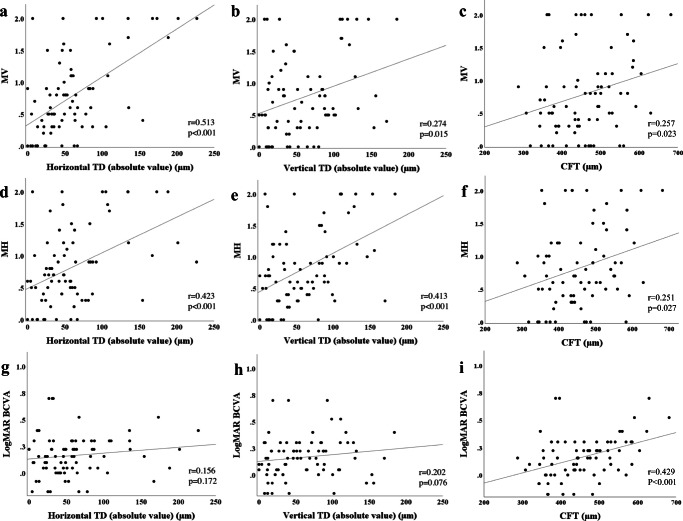
Scatter plots showing the relationship between the OCT parameters and visual function. (**a**) Relationship between the absolute value of horizontal “tangential displacement” (horizontal absolute TD) and vertical metamorphopsia score (MV). (**b**) Relationship between the vertical absolute TD and MV. (**c**) Relationship between the central foveal thickness (CFT) and MV. (**d**) Relationship between the horizontal absolute TD and horizontal metamorphopsia score (MH). (**e**) Relationship between the vertical absolute TD and MH. (**f**) Relationship between the CFT and MV (**g**) Relationship between the horizontal absolute TD and logMAR best-corrected visual acuity (BCVA). (**h**) Relationship between the vertical absolute TD and logMAR BCVA. (**i**) Relationship between the CFT and logMAR BCVA

The relationships between the OCT parameters and visual function are shown in Fig. 3. Regarding the TD, both MV and MH were correlated with horizontal absolute TD (MV: *r* = 0.513, *p* < 0.001; MH: *r* = 0.423, *p* < 0.001) and vertical absolute TD (MV: *r* = 0.274, *p* = 0.015; MH: *r* = 0.413, *p* < 0.001). However, there was no significant correlation between logMAR BCVA and the horizontal or vertical absolute TD. Conversely, CFT was correlated with all visual function measurements (MV: *r* = 0.257, *p* = 0.023; MH: *r* = 0.251, *p* = 0.027; logMAR BCVA: *r* = 429, *p* < 0.001) (Table 2). Multiple regression analysis showed that the horizontal absolute TD was an independent factor associated with both MV (β = 0.635, *p* < 0.001) and MH (β = 0.259, *p* = 0.048) (Table 3).

**Table 2:** Correlation of the absolute value of horizontal or vertical tangential displacement and central foveal thickness with visual function

	Horizontal absolute TD		Vertical absolute TD		CFT	
	*r*	*p*	*r*	*p*	*r*	*p*
MV	0.513	< 0.001*	0.274	0.015*	0.257	0.023*
MH	0.423	< 0.001*	0.413	< 0.001*	0.251	0.027*
LogMAR BCVA	0.156	0.172	0.202	0.076	0.429	< 0.001*

*MV* vertical metamorphopsia score, *MH* horizontal metamorphopsia score, *BCVA* best-corrected visual acuity, *TD* tangential displacement, *CFT* central foveal thickness

*Significant at *p* < 0.05 (Spearman’s correlation coefficient by rank test)

**Table 3:** Multiple regression analysis of OCT parameters associated with vertical and horizontal metamorphopsia score (MV and MH)

Dependent	Independent	*β*	*p*
MV	Horizontal absolute TD	0.635	< 0.001*
	Vertical absolute TD	− 0.148	0.237
	CFT	0.146	0.148
MH	Horizontal absolute TD	0.259	0.048*
	Vertical absolute TD	0.248	0.068
	CFT	0.116	0.285

*OCT* optical coherence tomography, *MV* vertical metamorphopsia score, *MH* horizontal metamorphopsia score, *TD* tangential displacement, *CFT* central foveal thickness; *β*, standard partial regression coefficient

*Significant at *p* < 0.05 (Spearman’s correlation coefficient by rank test)

## Discussion

The current study presents evidence about tangential morphological changes in the outer retina due to ERM and their correlation with metamorphopsia. We designed “TD” as a new parameter to quantify the tangential component of morphological changes in the outer retina. Our results showed that TD distribution had a tendency toward the temporal side from the central fovea along the horizontal meridian. The absolute TD was significantly correlated with metamorphopsia, but not correlated with visual acuity. We consider that the TD has the potential as indicator of metamorphopsia in patients with ERM.

The main cause of metamorphopsia is thought to be disrupted photoreceptor alignment [9]. With recent developments in retinal imaging technology, detailed reports of the relationship between metamorphopsia and retinal microstructure have emerged. Some of them confirm the classical hypothesis that morphological changes in the outer retina, which contains photoreceptors, are the major contributors to the origin of metamorphopsia. Ooto et al. found structural abnormalities in the photoreceptors, called microfolds, in eyes with ERM using adaptive optics scanning laser ophthalmoscopy, and this finding was associated with the degree of metamorphopsia [12]. Okamoto et al. reported a weak correlation between ONL plus OPL thickness and the degree of metamorphopsia in the eyes with ERM using SD-OCT [6]. Several reports have described the association between the outer retina status observed by OCT and metamorphopsia in the eyes with other macula diseases [13, 14]. Morphological changes in the inner retina have also been found to be associated with metamorphopsia. Several authors have reported a correlation between INL thickness and the degree of metamorphopsia using SD-OCT [3–7]. ERM is a representative vitreoretinal interface disease that affects the underlying retinal structures. Because the tangential traction force of the ERM primarily affects the inner retina, it is more likely that the inner retina, rather than the outer retina, is deformed by ERM. In that sense, morphological changes in the inner retina may be a useful indicator of the severity of traction force. Notably, the INL thickness was also reported as an indicator of VA in the eyes with ERM [15]. Kim et al. reported that the INL thickness was correlated with the degree of metamorphopsia and VA in the same eyes [5]. Moreover, Okamoto et al. indicated a weak correlation between ONL plus OPL thickness and VA in addition to metamorphopsia [6]. However, it has been reported that the degree of VA and metamorphopsia are not interdependent in patients with ERM [16]. This suggests that the underlying mechanisms of VA impairment and metamorphopsia may differ among the eyes with ERM. Although previous reports have shown that thickness measurements of the retinal layer on SD-OCT are good indicators of ERM severity, more specific indicators of metamorphopsia may exist. In this study, CFT was found to be associated with both BCVA and metamorphopsia. Conversely, we found no association between TD and BCVA, which was in contrast to metamorphopsia. Assuming that TD reflects the misalignment of photoreceptors more precisely, this result suggests that this parameter may be used as a specific indicator of metamorphopsia.

Metamorphopsia comprises different direction components of distortion. M-CHARTS consists of a series of dotted lines used to assess the component orthogonal to their axis. MV is determined by detecting the horizontal displacement of the dots in a vertical dotted line; therefore, it is assumed to correspond to the horizontal displacement of photoreceptors. Similarly, MH is assumed to correspond to the vertical displacement of photoreceptors. Arimura et al. reported the relationship between the horizontal contraction of the retina and MV and between the vertical contraction of the retina and MH by measuring the position movement of retinal vessels during the natural course of eyes with ERM in fundus photographs [17]. Ichikawa et al. followed this by measuring retinal vessel position movement in near-infrared images, before and after surgical removal of ERM [18]. These reports suggest that the direction of metamorphopsia depends on the direction of retinal morphological changes. In this study, Spearman’s rank correlation coefficient showed that the absolute value of horizontal TD correlated more strongly with MV than with MH, whereas the absolute value of vertical TD correlated more strongly with MH than with MV. Moreover, multiple regression analysis showed that the horizontal TD was an independent factor associated with MV. Regression coefficient indicated that the horizontal TD may be more responsible than other parameters for the vertical metamorphopsia. Certainly, the horizontal TD was also an independent factor associated with MH, but regression coefficient indicated there was no considerable difference between each parameter. One of the strengths of this study is that we focused on the outer retinal layer to evaluate tangential morphological changes of the retina by using SD-OCT. Our results support the hypothesis that the tangential distortion of photoreceptors causes metamorphopsia.

Naturally, the retinal structure is not symmetrical with regard to the fovea because of the presence of the optic disc and traveling of the retinal nerve fiber. In terms of retinal topographic changes caused by ERM, some asymmetric findings have been reported. For example, Ichikawa et al. reported that vertical retinal displacement was greater than horizontal retinal displacement [18]. More recently, Takagi et al. reported that the ONL around the fovea is naturally horizontally long and is more evident in patients with ERM [19]. In this study, SD-OCT measurements were performed in a small area restricted to the fovea. Our results revealed that the distribution of horizontal TD is inclined toward the temporal side from the fovea, whereas the distribution of vertical TD has no deviation. This result indicates that the ONL tends to be distorted toward the temporal side rather than the nasal side from the fovea. A possible hypothesis is that the optic disc is a restriction of distortion toward the nasal side from the fovea. Further studies are required to investigate the asymmetry of the foveal microstructure affected by ERM.

There were some limitations to our study. Our study was retrospective and included a relatively limited number of eyes. We evaluated only orthogonal two images along the horizontal and vertical meridian through the fovea, despite the retinal distortion actually occur in three-dimensions. Furthermore, the SD-OCT measurements were performed manually. Additionally, VA may have been affected by cataracts because most of the eyes were phakic at evaluation. From a methodological point of view, a possible ceiling effect may serve as a limiting factor for the establishment of correlation analysis, since the results of M-CHARTS have an upper limit. Future studies involving a larger sample size with automated computerized measurements and three-dimensional imaging are needed.

In conclusion, our study indicated that the outer retinal layer was distorted toward the temporal region from the central fovea by ERM. Furthermore, the tangential distortion of the outer retina was associated with the degree of metamorphopsia. Collectively, our results suggest that misalignment of parafoveal photoreceptors may cause metamorphopsia in patients with ERM.

## Data Availability

All data included in this study are available from the corresponding author upon reasonable request.
